# Characterization of a Heme-Regulated Non-Coding RNA Encoded by the *prrF* Locus of *Pseudomonas aeruginosa*

**DOI:** 10.1371/journal.pone.0009930

**Published:** 2010-04-08

**Authors:** Amanda G. Oglesby-Sherrouse, Michael L. Vasil

**Affiliations:** Department of Microbiology, University of Colorado Anschutz Medical Campus, Aurora, Colorado, United States of America; BMSI-A*STAR, Singapore

## Abstract

*Pseudomonas aeruginosa*, an opportunistic pathogen, requires iron for virulence and can obtain this nutrient via the acquisition of heme, an abundant source of iron in the human body. A surplus of either iron or heme can lead to oxidative stress; thus, the Fur (ferric uptake regulator) protein blocks expression of genes required for iron and heme uptake in iron-replete environments. Fur also represses expression of two nearly identical genes encoding the 116- and 114-nucleotide (nt) long PrrF1 and PrrF2 RNAs, respectively. While other Pseudomonads encode for the two PrrF RNAs at separate genomic loci, PrrF1 and PrrF2 are encoded in tandem in all sequenced strains of *P. aeruginosa*. In this report we characterize a third longer transcript encoded by the *prrF* locus, PrrH, which is repressed by heme as well as iron. We mapped the PrrH RNA in PA01 using 5′ rapid amplification of cDNA ends (RACE) and northern analysis, demonstrating the PrrH RNA is 325 nt in length. Accordingly, transcription of PrrH initiates at the 5′ end of *prrF1*, proceeds through the *prrF1* terminator and *prrF1*-*prrF2* intergenic sequence (95 nt), and terminates at the 3′ end of the *prrF2* gene. We also present evidence that repression of PrrH by heme causes increased expression of previously identified PrrF-regulated genes, as well as newly identified iron- and heme-activated genes. Thus, the PrrH RNA appears to impart a novel heme regulatory mechanism to *P. aeruginosa*.

## Introduction

*Pseudomonas aeruginosa* is a gram-negative opportunistic pathogen that causes serious infections in immuno-compromised individuals, such as burn victims and cancer patients, as well as persons with cystic fibrosis (CF). In order to cause disease, *P. aeruginosa* requires an abundance of iron, as evidenced by a multitude of studies [1], [2], [3], [4]. In anaerobic environments, iron in its ferrous form is freely diffusible through the outer membrane and transported into the cytoplasm by inner membrane transport systems. The insolubility of ferric iron in aerobic environments, however, limits accessibility to this nutrient, and the sequestration of iron by host proteins from potential pathogens creates a substantial barrier to infection. To scavenge insoluble or host-bound iron, many bacteria use siderophores, low molecular weight iron-chelating compounds. Ferri-siderophore complexes are bound at the cell surface by specific outer membrane receptors and transported into the periplasm, where a periplasmic binding protein delivers the complex to an inner membrane transporter [5]. Once in the cytoplasm, the siderophore is degraded, releasing the iron for use in a multitude of cellular processes including respiration, gene regulation, and environmental sensing [5].

*P. aeruginosa* synthesizes and secretes at least two siderophores, pyoverdin and pyochelin, both of which have been shown to be important for pathogenesis of *P. aeruginosa* [1], [4]. Although required for growth and virulence, the potential for iron-accelerated oxidative damage requires the uptake of iron and heme to be regulated in response to iron availability. In many gram-negative bacteria, including *P. aeruginosa*, this regulation is achieved by Fur (*f*erric *u*ptake *r*epressor), a 17-kDa iron-binding protein [6], [7]. Under iron-replete conditions, the Fur protein becomes ferrated and binds to a 19-bp Fur Box sequence in the promoters of genes required for iron and heme uptake, thereby preventing their transcription. In *P. aeruginosa*, Fur also affects the expression of several genes encoding virulence traits, including toxins and extracellular proteases [8], [9], [10]. Most Fur regulation in *P. aeruginosa* occurs through the repression of sigma factors, which in turn activate the expression of genes for siderophore biosynthesis and uptake. For example, Fur binds to the promoter and represses expression of *pvdS*, encoding a sigma factor that directly activates expression of genes for pyoverdin biosynthesis (*pvd*) and uptake (*fpv*), exotoxin A (*toxA*), and a secreted protease which degrades iron-binding proteins (*prpL*) [8], [11], [12], [13]. Binding of ferri-pyoverdin to its outer membrane receptor, FpvA, leads to activation of PvdS, which is normally sequestered at the inner membrane by its anti-sigma factor, FpvR [14], [15]. This paradigm of Fur-mediated regulation via sigma factors likely extends to the uptake systems for other iron sources.

**Figure 1:**
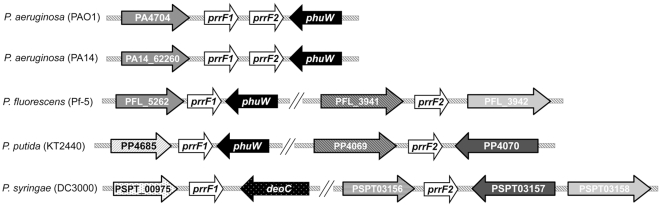
Genetic organization of *prrF* regions from different Pseudomonads. The tandem gene organization of *prrF1* and *prrF2* is restricted to *P. aeruginosa* strains. Block arrows indicate directionality of the open reading frame, and orthologous genes are represented by the color and pattern of the arrow. Map not drawn to scale.

In addition to genes for iron uptake and virulence, Fur represses the expression of two nearly identical genes encoding the PrrF1 and PrrF2 small regulatory RNAs, respectively [16]. The PrrF RNAs are functionally homologous to the RyhB RNAs encoded by *Escherichia coli*, *Shigella flexneri*, *Shigella dysenteriae*, and *Vibrio cholerae* [17], [18], [19], [20]. Additionally, iron-repressed homologs of PrrF have been identified in *Neisseria meningitidis* (NrrF) and *Azotobacter vinelandii* (ArrF) [21], [22]. In *E. coli*, RyhB binds to complementary sequences of target mRNAs, causing their degradation in an RNaseE- and Hfq-dependent manner [23], [24], [25]. RyhB can also stabilize at least one of its target mRNAs in *E. coli*, leading to its increased expression [26]. The most curious aspect of the PrrF RNAs is that they are encoded in tandem in *P. aeruginosa* strains, whereas all other sequenced Pseudomonads encode the two PrrF RNAs at distal genomic loci (Figure 1). Originally, the *prrF* locus of *P. aeruginosa* was thought to encode two differently-sized transcripts, with *prrF2* encoding an iron-repressed 111-nt RNA and *prrF1* encoding a 184-nucleotide (nt) RNA whose expression was affected by heme as well as iron [27]. More recently, the *prrF1 and prrF2* genes were shown to encode transcripts of similar size, approximately 110-nt in length [16]. Elucidation of the sequence, expression, and regulatory role of each of these transcripts should shed light on the *P. aeruginosa*-specific arrangement of the *prrF* genes.

Many pathogenic bacteria mediate the acquisition of iron via the uptake and degradation of iron-porphyrin, *i.e.* heme, an abundant source of iron in the human body. Although the contribution of heme acquisition to *P. aeruginosa* infection has not been studied in depth, heme uptake in other pathogens is known to play an integral role in infection [28], [29], [30]. *P. aeruginosa* mediates the uptake of heme via at least two systems: Phu (Pseudomonas heme uptake) and Has (heme assimilation system) [27]. The Phu system encodes for an outer membrane receptor that binds heme (*phuR*), a periplasmic heme binding protein (*phuT*), an inner membrane ATPase and permease (*phuUV*), and a cytoplasmic heme binding protein (*phuS*). The Has system, originally identified in *Serratia marcescens*, encodes for a secreted hemophore protein (*hasA*), which scavenges heme from hemoglobin [31], [32], and an ATPase and permease that are required for hemophore secretion (*hasDE*) [33]. An outer membrane receptor encoded by *hasR* binds the hemophore and extracts the heme, which is then transported across the outer membrane [34]. The imported heme is then bound by PhuT and transported into the cytoplasm by the Phu inner membrane permease system. A potential third outer membrane heme receptor is encoded by gene PA1302 in PA01; work is currently underway to determine the role of this gene in heme acquisition. PhuS binds imported heme in the cytoplasm, and is thought to traffic heme to at least one of two heme oxygenases expressed by *P. aeruginosa*, encoded by *hemO* [35] and *bphO* [36], which degrade the heme moiety, releasing biliverdin, carbon monoxide, and iron [37]. PhuS is also believed to act as a sensor of intracellular heme levels, contributing to the maintenance of heme and iron homeostasis [38]. Furthermore, while *hemO* is repressed by iron [35], expression of *bphO* is unaffected by iron [36], suggesting these two heme oxygenases are expressed under different environmental conditions. The biliverdin compounds generated by each heme oxygenase differ [35], [36], and *in vitro* studies have shown the direct delivery of heme by PhuS to HemO, but not BphO [39]. Thus, the functions of these two heme oxygenases in cellular physiology are likely distinct.

Heme itself is also able to mediate damage to cells via its hydrophobic quality and oxidative reactivity; yet, while the mechanism of Fur-mediated iron regulation is well understood, heme regulation in *P. aeruginosa* has not been studied in depth. The study discussed herein characterizes the *prrF*-encoded small RNAs, aiming to better describe their role in iron and heme regulation in *P. aeruginosa*. In this report, we confirm that the *prrF1* and *prrF2* genes encode PrrF RNAs of similar sequence and size, and we describe our identification of the sequence encoding a longer heme-regulated RNA, here named PrrH. Additionally we demonstrate heme regulates expression of PrrH, possibly via an anti-termination mechanism at the *prrF1* Rho-independent terminator. Furthermore, we begin to address the biological significance of the PrrH RNA and present evidence for its ability to mediate heme regulation of target mRNAs. Our findings yield new insights into the genetic organization of the *prrF1*, *prrF2*, and *prrH* genes in *P. aeruginosa* and introduce a new paradigm for heme regulation in gram-negative bacteria.

## Results

### The *prrF* locus encodes for a heme-regulated dimer of PrrF RNAs, here named PrrH

**Figure 2:**
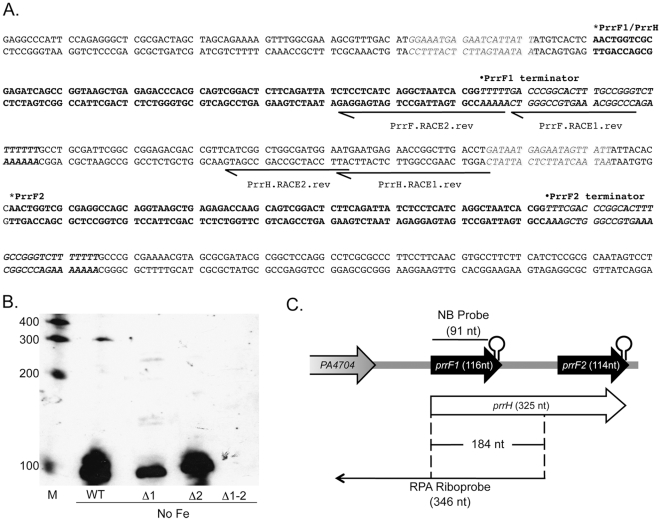
Identification of the PrrF and PrrH transcripts. A. Sequence of the *prrF* locus, showing the location of the transcriptional start sites for PrrF1, PrrF2, and PrrH determined by 5′ RACE as described in the Materials and Methods. The coding sequence of PrrF1 and PrrF2 is indicated by bold, the Fur binding sites preceding each *prrF* gene are indicated by italics, and the PrrF1 and PrrF2 Rho-independent terminators are indicated by bolded italics. B. Northern analysis of the PrrF and PrrH RNAs as described in the Materials and Methods, performed with RNA from PA01 wild type, Δ*prrF1*, Δ*prrF2*, and Δ*prrF1-2* strains grown for 18 hours in CM9 +1% glycerol. M – RNA size marker. C. Map of the *prrF* locus, showing the primer and probe binding sites for 5′ RACE, northern analysis, and previous RNase protection assays [25].

Our lab previously identified an apparent 184-nucleotide (nt) transcript originating from the *prrF* locus of *P. aeruginosa* strain PA01 by RNase protection assay (RPA) [27]. However, these assays did not allow for the *prrF*-encoded RNAs encoded by this region to be precisely mapped, which is necessary for understanding the regulation and function of each transcript. To determine the transcriptional start sites of the RNAs encoded by the *prrF* region, we performed 5′ rapid amplification of cDNA ends (RACE) using the primers PrrF.RACE1 and 2, designed to hybridize within the PrrF1 and PrrF2 RNAs as shown in Figure 2A. By this method, transcription of the PrrF1 RNA was found to begin where previously estimated [16], 116 nt upstream from the predicted Rho-independent terminator of PrrF1, while transcription of the PrrF2 RNA begins 114 nt upstream of its predicted Rho-independent terminator (Figure 2A). No other transcriptional start sites were detected upstream of PrrF1, suggesting that the longer *prrF*-encoded transcript is transcribed beyond the *prrF1* Rho-independent terminator. This idea was tested by 5′ RACE using primers that bind downstream of the *prrF1* terminator in the *prrF1-prrF2* intergenic region (Figure 2A - PrrH.RACE1 and 2), yielding a product that, when sequenced, extended from the confirmed transcriptional start site of the PrrF1 RNA. These data demonstrate that transcription of *prrF1* can continue through its putative Rho-independent terminator to generate a longer transcript.

Although 5′ RACE demonstrated where transcription of the longer *prrF*-encoded RNA begins, this technique did not clarify its 3′ sequence. Therefore, northern analysis was used to estimate the sizes of the *prrF*-encoded RNAs. Upon hybridization with a probe that should hybridize with both the PrrF1 and PrrF2 RNAs (shown in Figure 2C), three RNA species were apparent, two of which corresponded to the 116-nt and 114-nt PrrF1 and PrrF2 RNAs, respectively (Figure 2B). The third transcript was much longer than the anticipated size of 184 nt, migrating slower than the 300-nt marker band (Figure 2B). As was previously reported for the longer *prrF*-encoded RNA [27], this transcript was less abundant that the PrrF1 and PrrF2 transcripts (Figure 2B), and addition of either iron or heme to the growth medium eliminated detection of its expression by northern analysis (data not shown). The location of the riboprobe used for previous RPA analysis explains the apparent discrepancy in size estimation of this longer transcript: the distance from the PrrF1 transcriptional start site to the 5′ end of the RPA riboprobe is 183 nt (Figure 2C), just one nucleotide difference in length from what was previously reported to be the size of the longer transcript. Thus, it appears that this longer RNA is generated when transcription of PrrF1 continues through its Rho-independent terminator, extends through the *prrF1-prrF2* intergenic region (95 nt), and ends at the *prrF2* Rho-independent terminator (Figure 2C). Combined, these results indicate that the long PrrF transcript previously identified by RPA is actually 325 nucleotides in length and spans the entire *prrF* locus of *P. aeruginosa* (Figure 2C). Since this RNA is repressed by heme, we have named it PrrH for Pseudomonas RNA responsive to heme.

### Heme repression of PrrH does not require heme degradation

**Figure 3:**
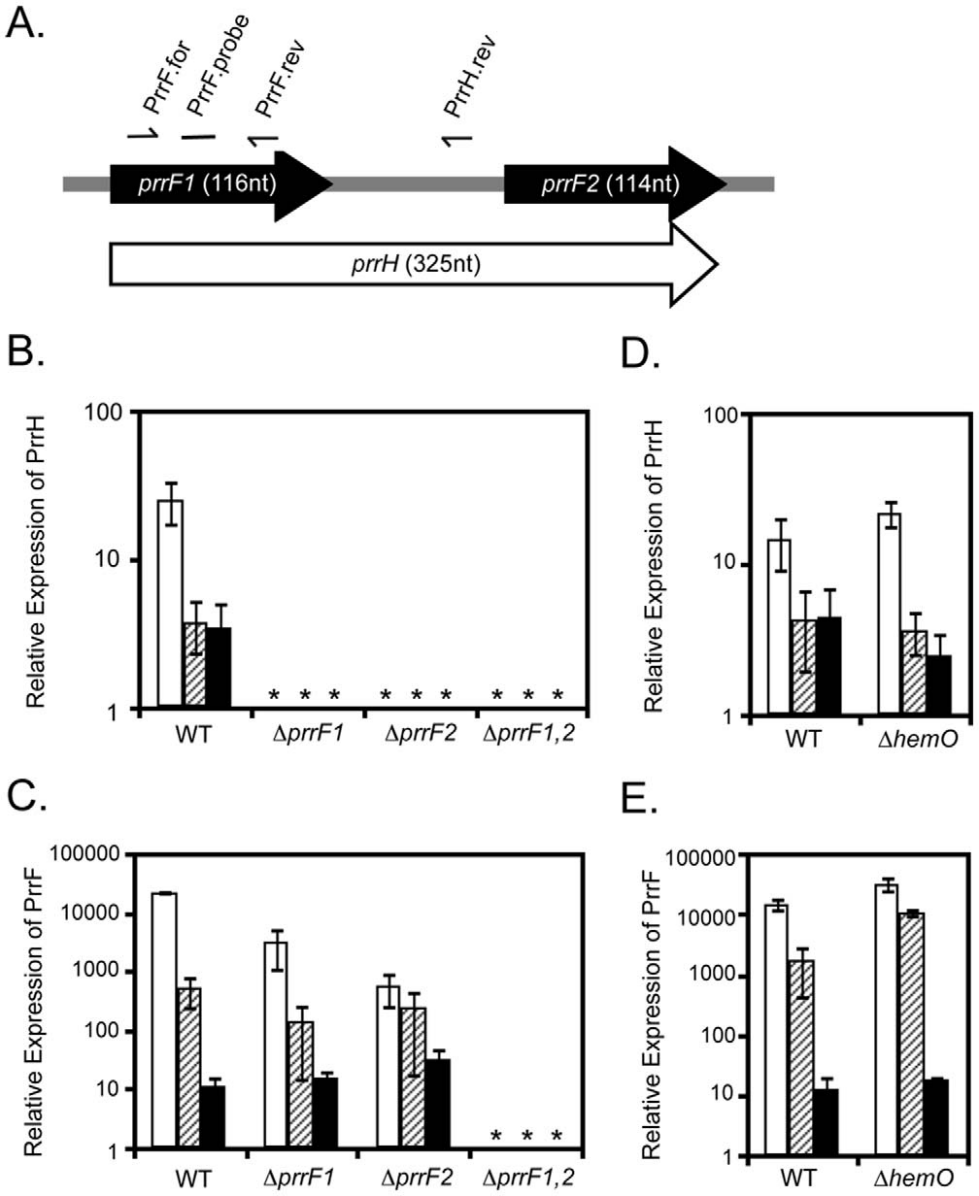
Quantification of PrrF and PrrH expression. A. Map of the PrrF-PrrH coding region showing the location of the primers and probes used for qRT-PCR. B-E. RNA was isolated from the indicated strains grown for 18 hours in CM9 +1% glycerol with no added iron (white bars), 40 µM hemin (hatched bars), or 100 µg/ml FeCl_3_ (black bars) and used for qRT-PCR analysis of the PrrH (B, D) and PrrF (C, E) RNAs as described in the Materials and Method. Error bars represent the standard deviation of three independent experiments. Asterisks (*) indicate expression was below detection levels.

In order to more thoroughly analyze PrrH expression, real time PCR (qRT-PCR) was employed to quantify the regulatory effects of iron and heme on the expression of the PrrF and PrrH RNAs. Primer pairs were designed to amplify the cDNA from all three *prrF*-encoded transcripts (prrF.for and prrF.rev) or the PrrH transcript specifically (prrF.for and prrH.rev), and a single probe was used to detect the PCR products amplified with either primer pair (Figure 3A). As expected, loss of either of the individual *prrF* genes eliminated PrrH expression (Figure 3B), while loss of the entire *prrF* locus was needed to eliminate PrrF expression (Figure 3C). Furthermore, addition of either iron or heme to our chelexed growth medium repressed PrrH expression (Figure 3B – [Fig pone-0009930-g004] [Fig pone-0009930-g005] [Fig pone-0009930-g006] [Fig pone-0009930-g007] 8.1-fold repression by iron and 7.2-fold repression by heme). In contrast, addition of protoporphyrin IX, an iron-free biosynthetic precursor of heme, to the growth medium caused no significant decrease in PrrH expression (data not shown), indicating repression by heme is not merely responsive to its porphyrin ring. Interestingly, heme also repressed PrrF expression (Figure 3C), possibly due to iron obtained from cytosolic heme degradation. Indeed, loss of the HemO heme oxygenase in a Δ*hemO* mutant reduced repression of PrrF by heme (Figure 3E), while, in contrast, causing a slight increase in heme repression of PrrH (Figure 3D). Residual heme repression of PrrF expression in the Δ*hemO* mutant is likely due to overlapping detection of the PrrH RNA by this primer-probe set, as well as effects from contaminating iron in the heme preparation. These data indicate that heme repression of PrrH is dependent on the entire heme moiety and distinguish heme regulation of the PrrH RNA from Fur-mediated iron regulation of the PrrF RNAs. Overall, our data indicate that expression of the PrrH RNA is repressed by iron, likely via the Fur protein, as well as heme via an unknown mechanism.

### PrrH is expressed maximally in stationary phase

**Figure 4 pone-0009930-g004:**
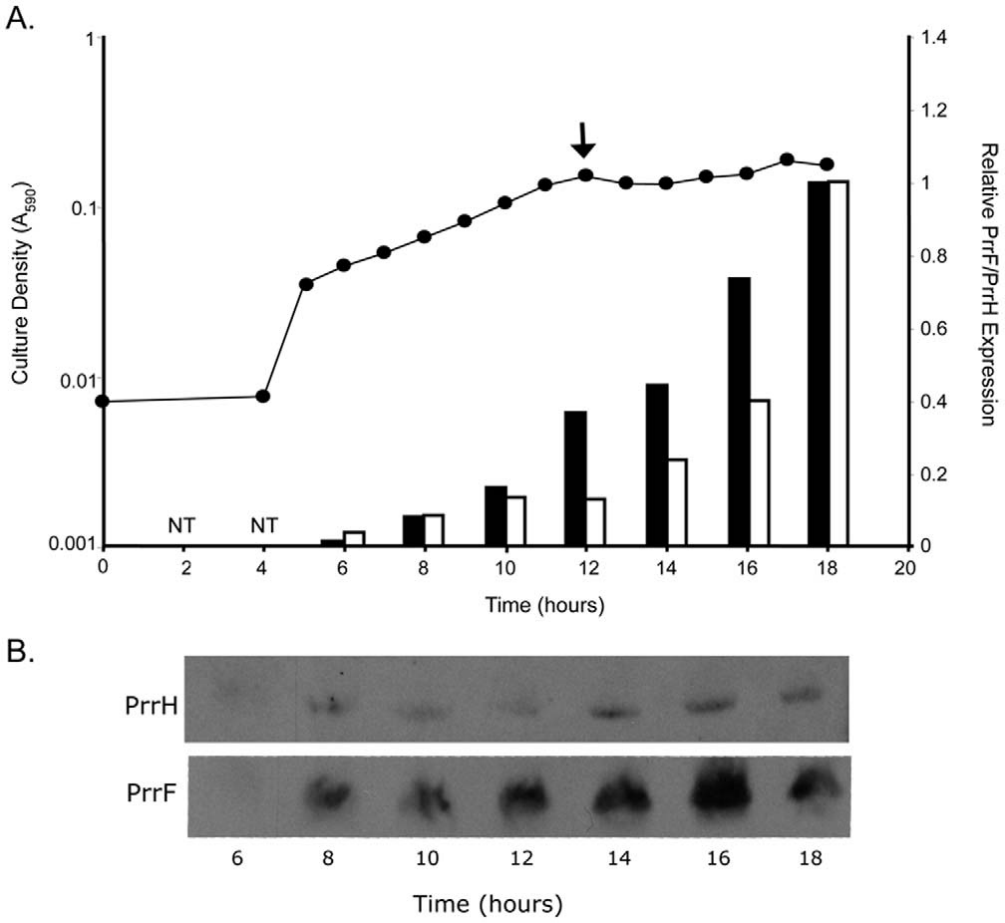
PrrF and PrrH are maximally expressed during stationary phase in iron-depleted medium. A. PA01 was grown in CM9 +1% glycerol for 18 hours, taking culture density (A_590_) measurements every hour. After the cells began growing logarithmically (∼5 hours) samples were taken for RNA isolation every two hours. Arrow indicates the onset of pyoverdin production. qRT-PCR analysis as described in the Materials and Methods was used to quantify PrrF (black bars) and PrrH (white bars) expression. B. RNA samples were also analyzed by northern blot as described in the Materials and Methods to detect the presence of the PrrF and PrrH RNAs.

Previous studies have shown that expression of most Fur-repressed genes in *P. aeruginosa*, including *prrF1* and *prrF2*, is maximal in stationary phase cultures grown in low iron medium, when intracellular iron stores have become depleted. Since expression of PrrH is repressed by iron, the current studies have concentrated on stationary phase cultures to look at PrrH expression. However, other regulatory mechanisms may cause this RNA to be most optimally expressed at different growth phases. To address this issue, the expression of PrrH at various phases of growth was determined. PA01 was grown under iron-depleted conditions for 18 hours, the time point normally used for PrrF expression analysis (Figure 4A). After entering logarithmic growth, the culture was sampled every two hours for RNA isolation, qRT-PCR and northern analysis. PA01 began secreting detectable amounts of pyoverdin (as measured by OD_420_) at a culture density of 0.15 (data not shown; indicated by an arrow in Figure 4A), at which point the growth rate decreased and the culture entered into stationary phase. This is likely when the intracellular iron stores of the cells were depleted, and pyoverdin production commenced in order to mediate iron acquisition. Expression analysis of PrrF and PrrH expression by both qRT-PCR and northern blot (Figure 4A-B) demonstrated that de-repression of these RNAs coincided with onset of pyoverdin production. This data is in agreement with our model that PrrF1 and PrrH are transcribed from the same Fur-repressed promoter, and that both are expressed optimally under iron-depleted conditions. These data also demonstrate that maximal expression of the PrrH RNA occurs, as previously indicated for PrrF, in stationary phase at approximately 18 hours of growth. Hence, we continued to use this time point for further analysis of PrrH expression.

### PrrF target genes are regulated by heme in a *prrF*-dependent manner

**Figure 5 pone-0009930-g005:**
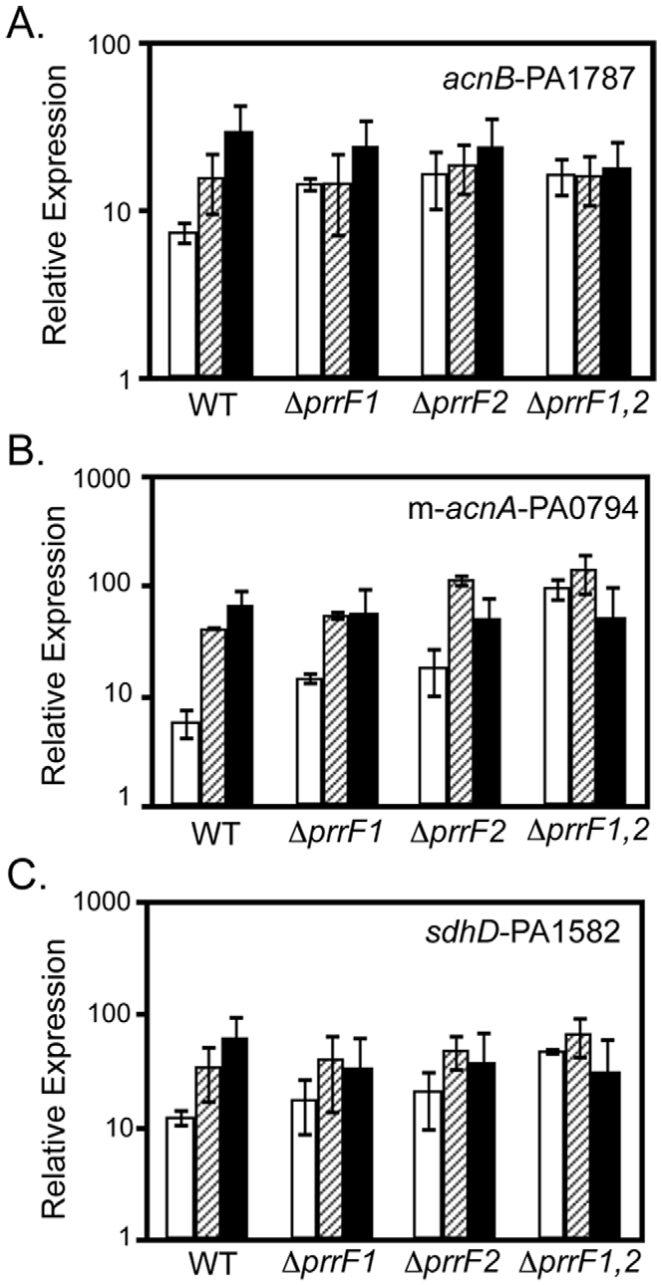
Effect of heme on the expression of PrrF-regulated genes. RNA isolated from the indicated strains, grown for 18 hours in CM9 +1% glycerol with no added iron (white bars), 40 µM hemin (hatched bars), or 100 µg/ml FeCl_3_ (black bars) was used for qRT-PCR as described in the Materials and Methods. Error bars represent the standard deviation of expression of (A) *acnB*-PA1787, (B) m-*acnA*-PA0794, and (C) *sdhD*-PA1582 from at least three independent experiments.

Previously, we identified greater than 50 genes as candidate PrrF-regulated genes by microarray and qRT-PCR analysis [16], [40]. These genes were identified as such when meeting the following three criteria: 1) induced by growth in high iron as compared to low iron media, 2) de-repressed in the Δ*prrF1-2* mutant as compared to wild type when grown in low iron media, and 3) unchanged in the complemented Δ*prrF1-2* mutant as compared to wild type in low iron media. Several of the genes identified by this analysis encoded metabolic enzymes, including succinate dehydrogenase (PA1581-4; *sdhCDAB*), aconitase A (PA1562; *acnA*), aconitase B (PA1787; *acnB*), and methyl-aconitase A (PA0794; m-*acnA*). To determine if the PrrH RNA was able to mediate repression of these genes in response to heme, the ability of iron and heme to affect their expression was examined by qRT-PCR. Similar to what was previously reported for iron [16], [40], expression of m-*acnA*, *acnB*, and *sdhD* was induced by heme in wild type PA01, although iron induction was stronger than heme induction for each of these genes (Figure 5A-C). These data may reflect the ability of iron to block expression of all three *prrF*-encoded RNAs, leading to complete de-repression of these target mRNAs, while addition of heme preferentially represses expression of the PrrH RNA, still allowing for some PrrF-mediated repression of these genes.

In order to test whether or not the PrrF and/or PrrH RNAs were responsible for the observed heme regulation of these target mRNAs, the regulatory effect of heme was examined in the *prrF* deletion mutants. As expected, deletion of either one of the *prrF* genes led to increased expression of *acnB*, m-*acnA*, and *sdhD* in low iron (Figure 5A-C), consistent with previous experiments performed in DTSB [16], [40]. Moreover, deletion of the entire *prrF* locus caused complete de-repression of these genes in low iron, eliminating the effects of iron and heme on their expression (Figure 5A-C). Interestingly, expression of m-*acnA* was de-repressed by deletion of *prrF2* in the presence of heme, while remaining unchanged in the presence of iron (Figure 5B), suggesting the PrrF2 RNA is expressed and represses expression of m-*acnA* in the presence of heme. Overall, these data demonstrate that heme mediates a regulatory effect on several previously-identified PrrF targets, and they suggest a role for either the PrrF and/or PrrH RNAs in this effect.

### The PhuR and HasR outer membrane heme receptors are important for heme-regulation of PrrH

**Figure 6 pone-0009930-g006:**
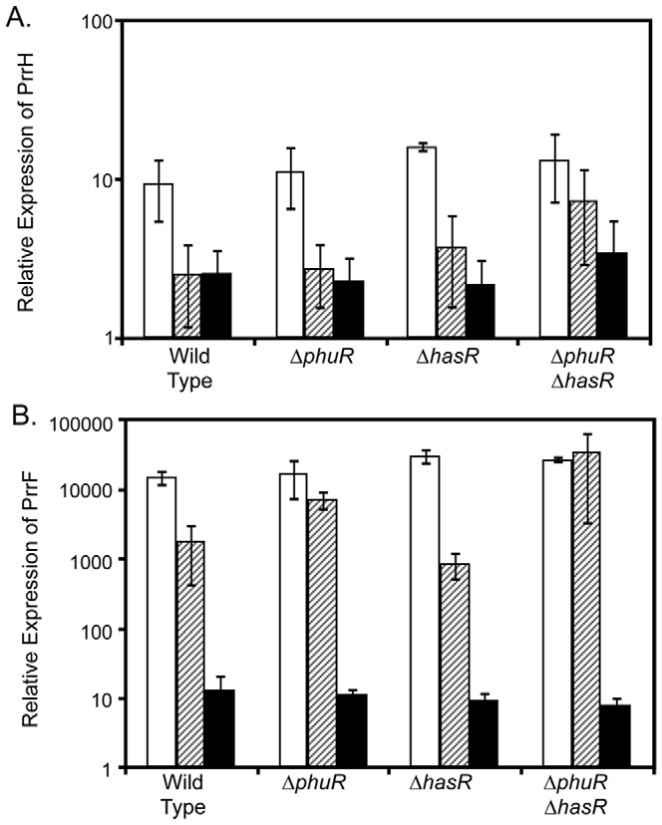
PhuR and HasR are important for heme regulation of PrrH. Heme utilization mutants lacking one or both outer membrane heme receptors were grown for 18 hours in CM9 +1% glycerol with no added iron (white bars), 40 µM hemin (hatched bars), or 100 µg/ml FeCl_3_ (black bars). RNA was then isolated and analyzed by qRT-PCR, as described in the Materials and Methods, for the mutants' ability to mediate heme and iron regulation of the PrrH (A) and PrrF (B) RNAs. Error bars show the standard deviation of at least three independent experiments.

Heme uptake is likely an important mechanism by which *P. aeruginosa* acquires iron, as it possesses at least two heme acquisition systems. Yet we know little of how this bacterium responds to heme as a signaling molecule. As one of the few examples of a heme-regulated gene in *P. aeruginosa*, the mechanism by which PrrH is regulated by heme is of immense interest. To better understand how this regulation occurs, we tested mutants lacking one or both of the known outer membrane heme receptors for their ability to mediate heme repression of PrrH. While loss of either PhuR or HasR alone had no effect on heme repression of PrrH, deletion of both heme receptors caused a small increase in PrrH expression in the presence of heme (Figure 6A). Residual heme repression of PrrH in the Δ*phuR*Δ*hasR* mutant, albeit statistically insignificant (*P*>0.2 by student's *t* test), could be mediated by a third putative heme outer membrane receptor encoded by PA1302; work is currently underway to determine the role of this gene in heme uptake and regulation. Since heme degradation is important for heme to have a regulatory effect on PrrF (Figure 3E), we hypothesized that PhuR and HasR would also be required for this regulation. In fact, loss of PhuR alone nearly eliminated heme repression of PrrF, and loss of both the PhuR and HasR heme receptors ablated the ability of heme to affect PrrF expression (Figure 6B). These data strengthen the idea that heme must be transported into the cytoplasm and degraded, releasing iron, in order to exert a regulatory effect on PrrF. Together, these data indicate that the PhuR and HasR heme receptors play a role in heme-regulated expression of PrrH. Furthermore, they demonstrate that heme repression of PrrH is mediated by a regulatory mechanism that is distinct from heme-dependent regulation of the PrrF RNAs.

**Figure 7 pone-0009930-g007:**
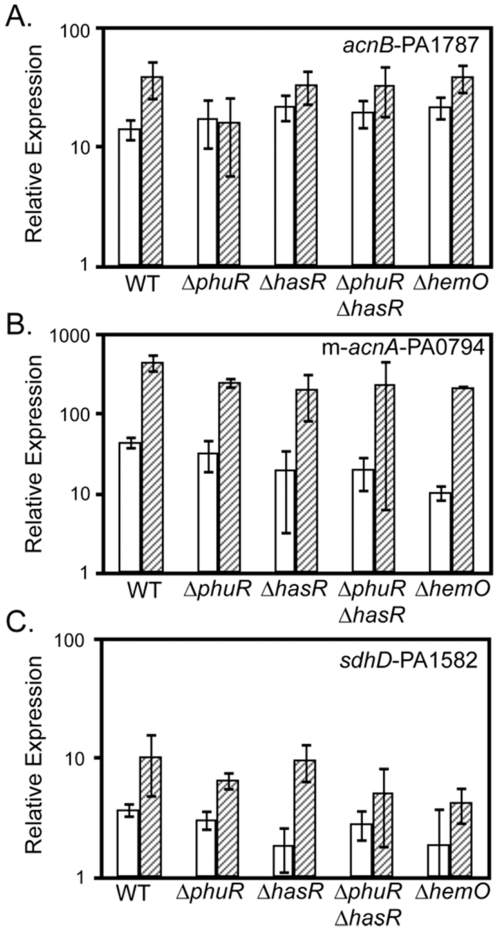
PhuR and HasR are important for heme regulation of PrrF target mRNAs. Heme utilization mutants lacking one or both outer membrane heme receptors or the HemO heme oxygenase were grown for 18 hours in CM9 +1% glycerol with no added iron (white bars) or 40 µM hemin (hatched bars). RNA was then isolated and analyzed by qRT-PCR for expression of (A) *acnB*-PA1787, (B) m-*acnA*-PA0794, and (C) *sdhD*-PA1582, as described in the Materials and Methods. Error bars show the standard deviation of at least three independent experiments.

Next, we sought to correlate the heme acquisition requirements of heme-regulated PrrH expression with that observed for previously-identified PrrF targets. For this, we tested the ability of heme to induce expression of *acnB*, m-*acnA*, and *sdhD* in our panel of heme utilization mutants. Simultaneous deletion of both the PhuR and HasR heme receptors caused noted defects in heme induction of both of *acnB* and *sdhD* (Figure 7A and C). Heme induction of *acnB* was impaired in the Δ*phuR* and Δ*hemO* mutants as well (Figure 7A); since heme is still capable of repressing expression of PrrH in the Δ*hemO* and Δ*phuR* mutants (Figures 3D and 6A, respectively), this data rules out a role for PrrH in repression of *acnB* expression. In contrast, heme induced expression of m-*acnA* in the single and double heme receptor mutants, although this induction was not statistically significant for the Δ*phuR*Δ*hasR* mutant due to variable m-*acnA* expression in the presence of heme (*P*>0.05 by student's *t* test; Figure 7B). Furthermore, deletion of *hemO* had no effect on heme induction of m-*acnA* (Figure 7B), providing the strongest evidence yet for regulation by the PrrH RNA. Results of this analysis for *sdhD* were less clear: while heme induction of *sdhD* was observed in the Δ*phuR* mutant, this induction was greatly affected by deletion of *hemO* (Figure 7C), possibly suggesting roles for both PrrH and PrrF in regulation of this target mRNA. Overall, these data suggest the PrrH RNA affects expression of m-*acnA*.

#### Regulation of a novel heme- and iron-regulated gene via PrrH

**Figure 8:**
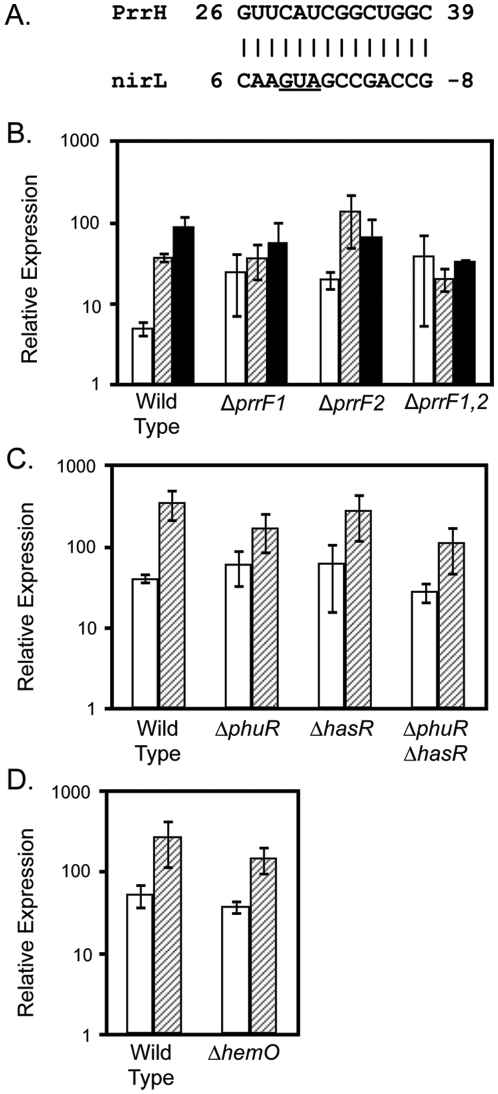
Expression of *nirL* is activated by heme in a PrrH-dependent manner. A. Complementarity between the PrrH unique sequence, derived from the *prrF1-prrF2* intergenic region, and the *nirL* mRNA. The underlined sequence indicates the start codon for *nirL* translation. B-C. RNA isolated from wild type PA01 and the (B) *prrF*, (C) heme receptor, and (D) heme oxygenase mutants, grown for 18 hours in CM9 +1% glycerol with no added iron (white bars), 40 µM hemin (hatched bars), or 100 µg/ml FeCl_3_ (black bars), was used for qRT-PCR as described in the Materials and Method. Error bars represent the standard deviation of expression from three independent experiments.

The unique sequence of PrrH derived from the *prrF1-prrF2* intergenic region offers the capability of this RNA to interact with and regulate the expression of a subset of mRNAs distinct from the PrrF regulon. We have identified several mRNAs that share complementarity with the unique sequence of PrrH by *in silico* analysis using Target RNA (http://snowwhite.wellesley.edu/targetRNA/). Among these were two genes related to heme biosynthesis: *nirL* (Figure 8A) and *thiE* (not shown). To begin defining the role of PrrH in heme regulation, these genes were selected for qRT-PCR analysis to characterize their ability to be regulated by iron and heme. While expression of *thiE* was unchanged by the addition of heme or iron (data not shown), expression of *nirL* was induced by both iron and heme (Figure 8B-D). Additionally, iron and heme induction of *nirL* were dependent upon the *prrF* locus (Figure 8B). Heme induction of *nirL* was reduced, but not eliminated, in the Δ*phuR*Δ*hasR* mutant (Figure 8C), while deletion of *hemO* caused no significant loss in heme induction of *nirL* (Figure 8D), indicating that heme regulation of this target mRNA is not due to iron from heme breakdown. Furthermore, no obvious complementarity was identified between the PrrF sequence and the operon containing *nirL*, suggesting heme regulation of *nirL* is not due to interaction of this mRNA with the PrrF RNAs. Overall these studies suggest a role for PrrH in heme activation of *nirL* and support a model in which PrrH regulates gene expression via its unique sequence derived from the *prrF1-prrF2* intergenic region.

## Discussion

Heme acquisition plays an important role in pathogenesis for several bacteria and is hypothesized to confer a similar advantage to *P. aeruginosa* during infection. While heme can serve as a valuable iron source in the host, free heme is toxic due to its hydrophobic quality and re-dox potential. Therefore, it is expected that a heme monitoring system coordinates the expression of genes for heme transport, degradation, and biosynthesis. Here we describe a non-coding RNA encoded by the *prrF* locus, named PrrH, and demonstrate that its expression is repressed by heme as well as iron. PrrH is the first example of a non-coding RNA that is encoded by two identical adjacent genes, each of which encode smaller regulatory RNAs. We have separately shown that the PrrH RNA is synthesized by other *P. aeruginosa* strains that share this genetic structure, including several clinical isolates (unpublished data). Additionally, PrrH is the first described non-coding RNA target of heme regulation in *P. aeruginosa* and potentially serves as the first example of a heme-responsive regulatory RNA in any bacteria. While the precise mechanism by which heme modulates PrrH expression remains unknown, our study has uncovered several requirements for this regulation. Notably, PrrF2 transcription still occurs from its own Fur-regulated promoter in the absence of the *prrF1* gene and promoter (Figure 2B), arguing against the idea that PrrF1 and PrrF2 are generated via processing of the longer PrrH RNA. Alternatively, our studies support a model in which PrrH expression occurs via anti-termination at the PrrF1 Rho-independent terminator.

Rho-independent terminators consist of a GC-rich palindromic element followed by a string of U residues, both of which are required for efficient termination. Transcription of the U oligonucleotide causes the RNA polymerase (RNAP) to pause, allowing time for the formation of a GC-rich hairpin structure in the nascent RNA [41]. The hairpin structure, along with the weak A-U interactions of the nascent RNA with template DNA, destabilize the transcription elongation complex (TEC), and both the RNA and DNA are released from the RNAP [41]. RNA-binding proteins can affect termination at sequences both upstream and downstream of the TEC, either by stabilizing the hairpin structure to increase termination efficiency, or by disrupting this structure and acting as anti-terminators [41], [42]. While the stem loops for Rho-independent termination of PrrF1 and PrrF2 transcription are identical, the sequences upstream and downstream of the PrrF1 and PrrF2 terminators vary (Figure 2A). These sequence variations are conserved in all sequenced *P. aeruginosa* strains and may provide a means for preferentially targeting a hypothetical PrrH regulatory protein to the PrrF1 terminator or PrrH unique sequence. Expression analysis of PrrH and PrrF in a series of heme utilization mutants indicates that heme regulation of PrrH is independent of promoter-centric regulation by Fur (Figures 3 and 6). Thus, our findings have led to a putative model in which heme regulates expression of PrrH via anti-termination of PrrF1 transcription. Future studies into the protein and sequence requirements for PrrH expression will be needed to fully understand the mechanism by which heme regulates expression of PrrH.

In several bacteria, proteins involved in heme acquisition provide feedback regulation of the genes for heme uptake, much like the FpvA pyoverdin receptor and PvdS sigma factor control expression of pyoverdin biosynthesis genes. Gram-negative organisms use ECF sigma factors responsive to heme uptake through the Has system in *Serratia marcescens* and the Bhu system in *Bordetella bronchispetica* to activate expression of heme uptake systems [43], [44]. Similarly, gram-positive bacteria utilize two-component regulatory systems (Chr and Hrr systems in *Corynebacterium diphtheriae* and Hss system in *Staphylococcus aureus*) to mediate heme-responsive regulation of their associated heme uptake systems [45], [46]. *P. aeruginosa* encodes for a putative ECF sigma factor and transmembrane sensor adjacent to the *hasR* gene, encoding one of the heme outer membrane receptors important for heme repression of PrrH. PA1302, encoding a putative outer membrane heme receptor, is also adjacent to a putative ECF sigma factor and transmembrane sensor. Thus, it is possible that interaction of heme with *P. aeruginosa*'s outer membrane heme receptors initiates a signaling cascade via one or both of these putative sigma factors, which in turn affect transcription of PrrH. Alternatively, periplasmic heme may be controlling the activity of an ECF sigma factor, possibly through interaction with an inner membrane anti-sigma factor, which ultimately leads to repression of PrrH transcription. Either scenario would allow heme to exert a regulatory affect on PrrH expression without transport into the cytoplasm, a necessity if cytoplasmic heme levels are relatively high already.

What is the biological function of PrrH? Our current model presumes that the PrrH regulon overlaps that of PrrF and includes oxidative stress protection, iron storage, and metabolic genes. Thus, the PrrF RNAs can repress these genes in response to iron, while PrrH can repress the same genes in response to either iron or heme. Expression data from analysis of the heme utilization mutants suggest the PrrH RNA is capable of repressing expression of at least one of these PrrF-regulated genes, m-*acnA*, under low iron conditions. Due to its unique sequence derived from the *prrF1-prrF2* intergenic region, PrrH is likely to regulate a specific subset of genes, possibly involved in heme biogenesis or other cellular processes. *In silico* analysis of this intergenic region using the TargetRNA application (http://snowwhite.wellesley.edu/targetRNA/) [47] has allowed for the identification of several putative PrrH-specific targets. We assessed the ability of heme and PrrH to regulate two of these targets and identified *nirL* as a novel iron- and heme-regulated gene. The *nirSMCFDLGHJEN* gene cluster encodes for dissimilatory nitrite reductase (NIR; cytochrome *cd*_1_) and includes genes for the biosynthesis of heme *d*_1_, a prosthetic group of NIR [48]. Biosynthesis of heme *d*_1_ branches from the central heme biosynthetic pathway, with NirF, NirJ, and NirE catalyzing its production from uroporphyrinogen III [49]. Thus, repression of NIR production by PrrH under limiting heme concentrations may prioritize the function of the heme biosynthetic pathway. While further work is needed to understand the contribution of the PrrH RNA to NIR expression, the data presented here suggest this RNA plays a role in heme regulation in *P. aeruginosa*.

This study has yielded insights into why *P. aeruginosa* has maintained the unique genetic structure encoding the PrrF1 and PrrF2 RNAs. That is, the tandem gene arrangement of the *prrF1* and *prrF2* genes seems to allow for heme-regulated expression of PrrF target mRNAs, which otherwise would only be regulated by iron, as well as genes unique to the PrrH regulon. Additionally, our study demonstrates that heme regulation of PrrH occurs by a mechanism that is distinct from heme regulation of PrrF. In the original study demonstrating heme-regulated expression of PrrH, a knockout of the entire *prrF* locus led to a significant decrease in growth on heme or hemoglobin as a sole iron source, suggesting a role for PrrH-repressed mRNAs in heme utilization [27]. Future work into the role of the PrrH RNA in overall cell physiology should elucidate the selective pressures for these regulatory phenomena.

## Materials and Methods

### Bacterial strains, growth conditions, and genetic manipulations

Bacterial strains used in this work are listed in Table 1. *E. coli* strains were routinely grown in Luria-Bertani (LB) broth or on LB agar plates, and *P. aeruginosa* strains were maintained in brain-heart infusion (BHI) broth or on BHI agar plates. For a defined high and low iron medium, a chelexed M9 (CM9) medium was developed by treating 10X M9 salts [50] with chelex and supplementing with 1% glycerol. FeCl_3_ was added to a final concentration of 100 µg ml^−1^ as indicated; hemin and protoporphyrin IX were added to a final concentration of 40 µM. Antibiotics were used at the following concentrations (per milliliter): 100 µg of ampicillin, 15 µg of gentamicin, and 15 µg of tetracycline for *E. coli* and 750 µg of carbenicillin, 75 µg of gentamicin, and 150 µg of tetracycline for *P. aeruginosa*. The Δ*hemO*::*gm* mutant was generated by amplifying the altered *hemO* fragment from IA614 [35]. This fragment was cloned into PCR2.1 (Invitrogen), then sub-cloned into pEX18Tc [51] (pEX-*hemO::gm*). The resulting plasmid was conjugated from SM10 *λpir* [52] into PA01, and mutants were selected on gentamicin. Resolved mutants were isolated on sucrose and confirmed for loss of the plasmid backbone by lack of growth on tetracycline. The final mutant was confirmed by PCR of the *hemO* region.

**Table 1:** Bacterial strains used in this study.

Strain	Description	Source/Reference
PA01	Wild type *P. aeruginosa* strain; originally isolated from a human wound in 1955 in Australia	[53]
Δ*prrF1*	Deletion mutant of *prrF1* in PA01	[16]
Δ*prrF2*	Deletion mutant of *prrF2* in PA01	[16]
Δ*prrF1-2*	Deletion mutant of entire *prrF* locus in PA01	[16]
Δ*phuR*::*gm*	Deletion mutant with *phuR* gene replaced by gentamicin cassette in PA01	[27]
Δ*hasR*::*tc*	Deletion mutant with *hasR* gene replaced by tetracycline cassette in PA01	[27]
Δ*phuR*Δ*hasR*	Δ*phuR*::*gm* mutant with *hasR* gene replaced by tetracycline cassette in PA01	[27]
Δ*hemO*::*gm*	Δ*hemO*::*gm* mutation from strain IA614 [35] moved into the wild type PA01 background	This study
DH5α	*E. coli* cloning strain; *endA1 hsdR17 supE44 thi-1 recA1 gyrA relA1* Δ (*lacZYA-argF*)U169 *deoR* [ϕ80 dLacΔ (*lacZ*)M15]	[50]
SM10 *λpir*	*E. coli* strain used for conjugation; *pir*R6K	[52]

### 5′ rapid amplification of cDNA ends (RACE)

5′ RACE (Invitrogen) was used to identify the transcriptional start sites of PrrF and PrrH as described in the kit's instructions. Briefly, RNA was isolated from PA01 grown in iron-depleted conditions on RNeasy Mini Columns (Qiagen), and cDNA was generated using PrrF.RACE1 (CCCGGCAAAGTGCCGGGTC) or PrrH.RACE1 (CAGGTCAAGCCGGTTCTCATTCAT). The cDNA was tailed with dCTP using terminal deoxy-transferase, and a poly-G primer (Invitrogen) was combined with either PrrF.RACE1 or PrrH.RACE1 for PCR. A second PCR was carried out with a dilution of the first PCR, using nested primers – PrrF.RACE2 (AAAACCGTGATTAGCCTGATGAGGAG) or PrrH.RACE2 (ATTCCATCGCCAGCCGATG) – with an adaptamer primer provided by Invitrogen. The PCR products from this reaction were analyzed by gel electrophoresis, and predominant products were purified, cloned into PCR2.1 and analyzed by sequencing.

### Real time PCR

Strains were grown at 37°C for 18 hours in CM9 or DTSB and supplemented with the indicated amounts of FeCl_3_ or hemin. Total RNA was isolated on RNeasy Mini Columns and DNase-treated with RNase-free DNaseI (New England Biolabs). cDNA was prepared from 50 ng of RNA using the ImProm II Reverse Transcription System (Promega). Real time PCR reactions were carried out in a LightCycler® 480 using the LightCycler® 480 RNA Master Hydrolysis Probes master mix (Roche), and data was analyzed using the LightCycler® 480 software. Relative amounts of cDNA were normalized by dividing the expression values by the relative amounts of *omlA* cDNA in each sample.

### Northern blots

For northern analysis, 10–20 µg of total RNA isolated on RNeasy Mini Columns was run on a 6% polyacrylamide denaturing (7M urea) gel then transferred to a BrightStar® membrane (Ambion) using a semi-dry transfer apparatus. A biotinylated probe to *prrF1* was generated by PCR amplification using the following primers: PrrF1.NB.for (CGCGAGATCAGCCGGTAAGC) and PrrF1.NB.rev (GTGCCGGGTCAAAAACCGTG). The probe was heat-denatured, labeled using the BrightStar® Psoralen Biotin nonisotopic labeling kit (Ambion), and hybridized to the blot overnight at 42°C. The membrane was washed using the Ambion Northern Max Low Stringency and High Stringency wash solutions according to the manufacturer's instructions. Detection of the biotinylated probes was carried out using the BrightStar® BioDetect™ nonisotopic detection kit (Ambion).
